# Analysis of the Overlapped Electrochemical Signals of Hydrochlorothiazide and Pyridoxine on the Ethylenediamine-Modified Glassy Carbon Electrode by Use of Chemometrics Methods

**DOI:** 10.3390/molecules24142536

**Published:** 2019-07-11

**Authors:** Yue Zhang, Yan Zhou, Shujun Chen, Yashi You, Ping Qiu, Yongnian Ni

**Affiliations:** 1 Port of Caofeidian Group Co., Ltd., Tangshan 063200, China; 2 College of Medicine, Nanchang University, Nanchang 330031, China; 3 Department of Chemistry, Nanchang University, Nanchang 330031, China

**Keywords:** differential pulse voltammetry, hydrochlorothiazide, pyridoxine, chemometrics

## Abstract

In this work, the electrochemical behavior of hydrochlorothiazide and pyridoxine on the ethylenediamine-modified glassy carbon electrode were investigated by differential pulse voltammetry. In pH 3.4 Britton-Robinson (B-R) buffer solution, both hydrochlorothiazide and pyridoxine had a pair of sensitive irreversible oxidation peaks, that overlapped in the 1.10 V to 1.20 V potential range. Under the optimum experimental conditions, the peak current was linearly related to hydrochlorothiazide and pyridoxine in the concentration range of 0.10–2.0 μg/mL and 0.02–0.40 μg/mL, respectively. Chemometrics methods, including classical least squares (CLS), principal component regression (PCR) and partial least squares (PLS), were introduced to resolve the overlapped signals and determine the two components in mixtures, which avoided the troublesome steps of separation and purification. Finally, the simultaneous determination of the two components in commercial pharmaceuticals was performed with satisfactory results.

## 1. Introduction

Hypertension is an independent disease characterized by blood pressure above the normal range, and its etiology is not yet clear. According to the World Health Organization (WHO), hypertension is defined as adult blood pressure exceeding 21.3/12.6 kPa (160/95 mmHg) [1,2]. As a common cardiovascular disease, the risk of hypertension is not only revealed in high blood pressure, but also in a variety of pathophysiological changes, including changes in cardiovascular structure and function, nephropathy, encephalopathy and retinopathy, causing coronary atherosclerosis, cerebrovascular sclerosis and ultimately life-threatening [3,4,5]. There are an estimated one billion cases of hypertension in the world, which is predicted to rise to over 1.5 billion by 2015 [6,7]. In China economic development has changed people’s habits and diet, the elderly population has grown and the incidence of hypertension has risen to 27.8% [2,8]. Clinical studies have found that reasonable drug treatment can effectively control blood pressure and various symptoms [9,10]. Moreover, it can reduce the incidence of some related diseases, thereby improving people’s quality of life [11].

Among hypertensive patients, it is found that a single antihypertensive drug cannot lower blood pressure to normal, so two or more antihypertensive drugs are necessary to make blood pressure normal [12,13,14]. Each of the drug components of compound medicines have synergistic effect, which not only enhances the antihypertensive effect, but also counteracts the adverse side effects [15,16,17]. Most compound antihypertensives contain the diuretic-antihypertensive drug hydrochlorothiazide and a small amount of vitamin B_6_ (pyridoxine) to protect the heart and prolong the antihypertensive effect.

Hitherto, various analytical methods have been reported for the separation and determination of the components in compound preparations. It has been reported that HPLC can be used in combination with various detection techniques, such as electrochemical detection [18], mass spectrometry [19,20], desorption electrospray ionization [21], size-exclusion chromatography [22], chemiluminescence [23], or spectrophometry [24]. The electroanalytical chemistry method, with its advantages of higher sensitivity and selectivity, is preferable, and it has become a common method for drug analysis [25]. In electrochemistry, there are sereval ways to avoid overlapping signals: (1) selection of an appropriate pH and electrolyte; (2) analyte derivation; and (3) application of chemometrics. Among them, statistical chemometrics methods are environmentally friendly, and can avoid the troublesome steps of separation and purification. However, complex background currents often exist in voltammetric and polarographic analysis of drugs. The oxidation or reduction processes of electrodes are often irreversible, and their peak potentials will change with the change of drug concentration. This non-linear additive system will have a serious impact on the determination [26,27,28]. Moreover, it is extremely difficult to directly measure the content of a single component with serious overlap in a complex mixture. Therefore, the application of chemometrics in pharmaceutical electroanalytical chemistry has attracted extensive attention [29]. Classical least squares (CLS), principal component regression (PCR) and partial least squares (PLS) are commonly used [30].

In this work, the differential pulse voltammetry technique was used to investigate the electrochemical behavior of hydrochlorothiazide and pyridoxine on an ethylenediamine-modified glassy carbon electrode. The fabricated electrode has good stability and reproducibility [31,32], and the modified version has better electrochemical charateristics [33]. Their overlapping peaks were resolved by chemometrics, which avoided the tedious step of separation and purification, simplified the determination process and realized the simultaneous determination of two components in commercial pharmaceuticals.

## 2. Results and Discussion

### 2.1. Voltammetry at a Modified Electrode

**Figure 1:**
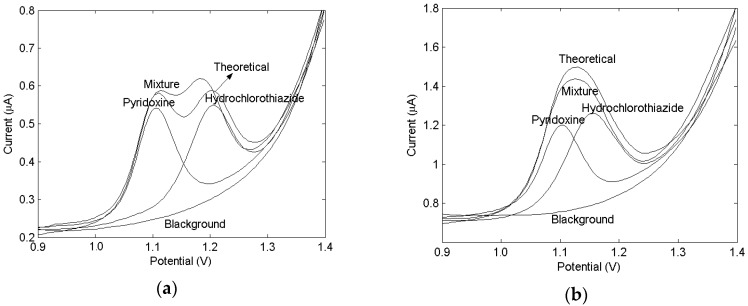
The differential pulse voltammograms (DPV) of 0.4 μg/mL hydrochlorothiazide and 0.2 μg/mL at the ethylenediamine-modified glassy carbon electrode (**a**) and bare glassy carbon electrode (**b**) respectively in the pH 3.4 B-R buffer.

Figure 1 shows the differential pulse voltammograms of 0.4 μg/mL hydrochlorothiazide and 0.2 μg/mL pyridoxine at the ethylenediamine-modified glassy carbon electrode (Figure 1a) and bare glassy carbon electrode (Figure 1b), respectively, in pH 3.4 B-R buffer. 

The voltammetric curves of each compound and their mixtures show the maximum peak potentials for hydrochlorothiazide and pyridoxine at 1.11V and 1.22V, respectively, as well as the heavily overlapped nature of the composite voltammograms of the mixtures. This indicated that the sensitivity of the two drugs on the modified electrode was stronger than that on the bare electrode, and the peak potential difference between the two drugs is larger, which was helpful for chemometric analysis.

Ethylenediamine modifies the surface of the glassy carbon electrode by covalent bonding, which is based on oxidation of amino groups [34,35]. Primary and secondary amines can form amine radical cations through the oxidation of the amino group in anhydrous ethanol or acetonitrile solution. The radical is further bonded on the surface of glassy carbon or carbon fibers to form C-H covalent bonds [36,37]. Therefore, the modification method is also called the “amine radical cation method”.

It is generally believed that the modifying cation and the electrode are vertically oriented. Primary amines are easily grafted on the surface of electrode during the electrooxidation, while secondary amines oxidize to form a monolayer with low coverage, and tertiary amines cannot form a monolayer. Therefore, it can be concluded that the steric hindrance of the substituent affects the active site of radicals on the surface of the electrode during the amine oxidation process. The bonding reaction requires protons to be detached from the amine cation radical, so this reaction path is not feasible for tertiary amines [38]. Ethylenediamine is a typical aliphatic diamine, which exhibits characteristics of both primary and secondary amines. The chemical properties of ethylenediamine are related to the number of hydrogen atoms substituents on the functional group (NH_2_). In this experiment, it seems the special chemical structure coordinates on the surface of the electrode to form an ethylenediamine-modified electrode.

As for the repeatability of the modified electrode, the results showed that the relative standard deviation (n = 3) was less than 3.8%. Regarding stability, samples containing the drugs were stored in the refrigerator at 4 °C. After 15 days, the current was measured with a standard deviation of less than 3.4%. The results proved that the modified electrode was stable and the processes have good repeatability.

### 2.2. Selection of Buffer Solution

This experiment investigated the effects of various buffer solutions on the electrochemical response of drugs. The tartaric acid-sodium tartrate buffer, acetic acid-sodium acetate buffer, disodium hydrogen phosphate-citric acid buffer and Britton-Robinson buffer were investigated. It was found that the sensitivity and symmetry of the oxidation peaks of each component in the Britton-Robinson buffer were highest, so the B-R buffer was selected.

The effect of acidity on the voltammetric curves of the drugs was also investigated. The electrochemical voltammetric curves of hydrochlorothiazide and pyridoxine in a series of B-R buffer solutions at pH 1.98–11.98 were measured, respectively. It was found that the peak current of the drugs increased and then decreased with the increase of pH value, and the peak shape gradually broadened. The peak potential of the drugs moved negatively with the increase of pH and showed a linear relationship. The linear equations were expressed as below:

Hydrochlorothiazide: *E*_p_ = −0.0468 *pH* + 1.3202 (*R* = 0.999), (1)

Pyridoxine: *E*_p_ = −0.0515 *pH* + 1.2710 (*R* = 0.998), (2)

In acidic B-R buffer (pH 3.4), the drugs had better peak shape and higher peak sensitivity. Thus, these results clearly indicated that the pH 3.4 B-R buffer solution should be selected as supporting electrolyte in this experiment.

### 2.3. Cyclic Voltammetry to Study the Adsorptivity of the Electrode Reaction

**Figure 2:**
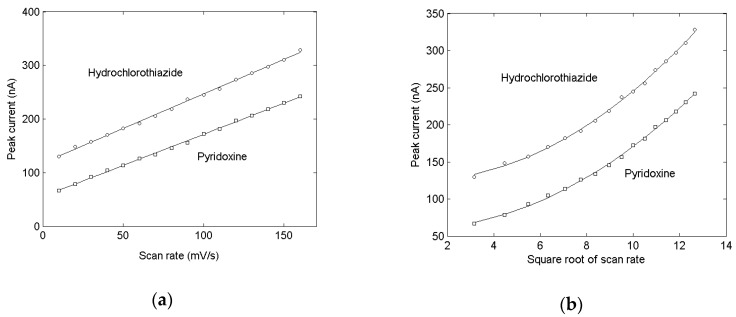
Effect of scan rate (**a**) and square root of scan rate (**b**) on peak current of the drugs.

The effect of the scan rate and the square root of the scan rate on peak current by cyclic voltammetry for the different drugs is examined (Figure 2). The peak current of both components showed a linear relationship with the change of scan rate, but had a upward bending curve with square root of scan rate, illustrating that the electrode process is controlled by the adsorption rate at that time.

### 2.4. Linearity Ranges and Limits of Detection for Drugs

**Figure 3:**
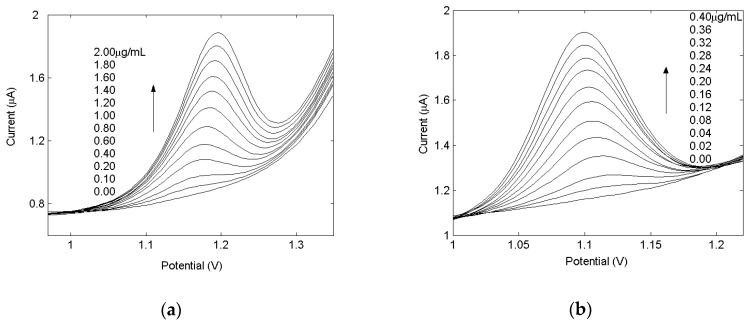
Voltammogram of hydrochlorothiazide (**a**) and pyridoxine (**b**) for different concentrations.

Under the optimal experimental conditions, the drug concentrations had linear relationships with the electrochemical signals. Figure 3 shows voltammetric curves for the determination of hydrochlorothiazide (a) and pyridoxine (b). There is a good linear relationship between the peak current and the drugs concentration in the range of 0.02–0.40 μg/mL for pyridoxine and 0.10–2.0 μg/mL for hydrochlorothiaxide. The regression parameters are summarized in Table 1, and the accuracy of the determination of these drugs has also been established by analysing the minimum concentration calibration graph for six known solutions, i.e., 0.02 μg/mL (see Table 1).

The relative standard deviation (R.S.D.) values obtained were 1.7% and 2.1% for pyridoxine and hydrochlorothiazide, respectively, and the detection limit values were 7.00 and 37.60 ng/mL for pyridoxine and hydrochlorothiazide, respectively. Therefore, it can be concluded that the proposed electrochemical analysis method for the determination of individual drugs is reliable.

### 2.5. Prediction of Synthetic Mixtures of Drug Compounds

Before the determination of unknown mixtures by chemometrics, a set of mathematical models should be developded to correct the concentration of each component in the mixtures. According to the four-level orthogonal array design represented by OA_16_(4^5^), a set of standard samples was prepared, which indicated that a data set of 16 samples was required. The calibration set is based on the given concentration range for 0.02–1.0 μg/mL, as it is usually desirable to test the lower performance of the calibration rather than the higher concentrations [39]. The prediction power of the calibration model was then assessed using another set of samples consisting of 12 synthetic mixtures. In our work, CLS, PLS, and PCR models were established and their prediction errors were compared. CLS method is often called K matrix method, which is a commonly used multivariate correction method. This method is based on multiple linear regression and is frequently used for quantitative voltammetric analysis, which was a much common multivariate calibration method [40]. PCR and PLS, which are powerful multivariate statistical tools and are available as commercial software for laboratory computers [41], are based on factor analysis [42]. These methods have many of the full-soectrum advantages of the CLS method and have also been successfully applied for voltammetric analysis [43].

**Figure 4:**
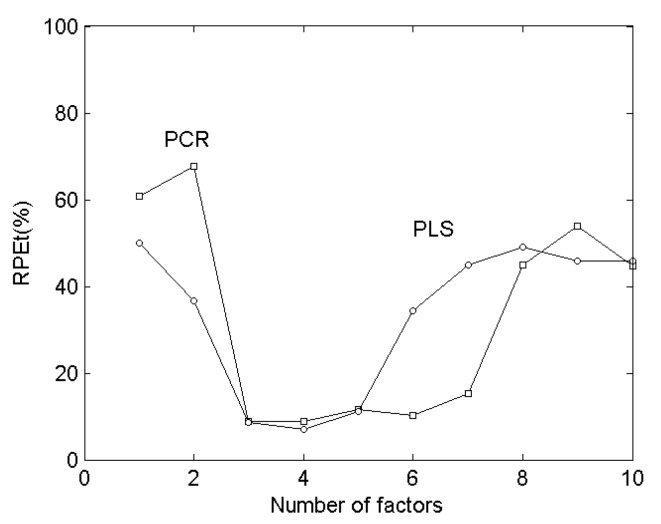
The relationship of PRE_t_ and the number of factors used for PLS and PCR.

From the measured results in Table 2, the best prediction results could be obtained by the PLS method, while the CLS method was the worst. Because of the background current and the interaction between components in voltammetric analysis, the non-linear superposition of voltammograms would occur and the CLS method had some difficulties in analyzing such systems, while PCR and PLS are calibration methods based on factor analysis, which could better solve this kind of non-linear problem [44]. In the PCR and PLS methods, a certain number of factors must be selected to perform a matrix factorization, so that the response matrix could be reproduced within the experimental error range. The selection of factors has a great impact on the calculation results. Generally, when the number of factors was less than or slightly larger than the component number, the relative standard deviation of the calculation results was smaller [45]. Figure 4 shows the relationship between the relative error and the number of factors. In this way, when the PCR method was used, the number of factor was 4, the relative error was smaller and the relative error is the minimum when the number of factor was 4 by PLS method.

## 3. Detection of the Drugs in Real Samples

The proposed method was applied for the determination of the drugs in four commercial tablets, produced by two different pharmaceutical companies. In each case, the sugar coating was removed from ten tablets which were then ground into a powder and dissolved in doubly distilled water. After filtering three times the filtrate was collected and diluted to 50 mL with distilled water.

According to the described PLS procedure, the samples were analyzed with the results listed in Table 3. The values measured by the proposed method were consistent with the target values. The average relative error between two methods was 3.1%, indicating the method provided excellent precision in detecting the drugs. The recovery rate was between 92.5% and 106.2%, with RSD (n = 5) less than 2.9%. It could be seen that the simultaneous determination of hydrochlorothiazide and pyridoxine in compound medicines could be achieved by the proposed electrochemical method without labelling, and the results were satisfactory.

## 4. Materials and Methods

### 4.1. Reagents and Apparatus

Hydrochlorothiazide (0.1 mg/mL) and pyridoxine (0.02 mg/mL, ethanol) were obtained from Shanghai Biochemical Reagent Co. Ltd. (Shanghai, China). Ethylenediamine (>99%) was dissolved to 1 mol/L solution with anhydrous ethanol for use as the ethylenediamine-ethanol modifier. A Britton-Robinson (B-R) buffer solution of pH 3.4 was prepared by adding 15 mL 0.2 mol/L sodium hydroxide into 100 mL of a mixed acid containing 0.04 mol/mL each of orthophosphoric acid, acetic acid, and boric acid.

All chemicals used (Sinopharm Chemical Reagent Co., Ltd. Shanghai, China) were of analytical-reagent grade and all the solutions were prepared with doubly distilled water. The experiments were carried out at room temperature.

A CHI 660A electrochemical workstation (Shanghai CH Instruments Co., Shanghai China) equipped with a BAS C-1 cell stand was used for voltammetric measurements. The three electrode system consisted of a glassy carbon electrode as work electrode, Ag/AgCl reference electrode and platinum wire as auxiliary electrode. The pH measurements were performed on a SA720 meter (Thermo Orion, Waltham, MA, USA).

### 4.2. Procedure

#### 4.2.1. Modification of Glassy Carbon Electrode

Firstly the glassy carbon electrode was polished on 1200-grit Carbimet metallographic sandpaper, and to a mirror finish with 1.0 μm α-alumina powder, 0.3 μm α-alumina powder and 0.05 μm γ-alumina powder in turn. Then HNO_3_ (1:1), anhydrous ethanol and doubly distilled water were used to clean the electrode with ultrasonic irradiation, respectively. Finally, the activated electrode was dipped in 1 mol/L ethylenediamine-ethanol solution for 12 h and the ethylene- diamine-modified glassy carbon electrode was ready for use.

#### 4.2.2. Procedure

A suitable amount of standard solution of hydrochlorothiazide and pyridoxine or their mixed solution were prepared in the electrolytic cell, together with B-R buffer (2 mL pH 3.4). The solution was diluted to 10 mL with doubly distilled water and shaken evenly. Then the ethylenediamine-modified glassy carbon electrode was placed, the solution was thoroughly mixed by stirring for 15 s. After a 10 s static period, a differential pulse voltammetric scan was run from 0.9 to 1.4 V. The resulting voltammograms were sampled by a computer at 4 mV intervals. The experiment was completed at 20 °C room temperature.

## 5. Conclusions

In this work, the use of a modified electrode to improve the selectivity of voltammetric data in complex matrices is the issue, even if the separation is not complete and requires the use of chemometric tools. Furthermore, PCL and PCR seem to perform adequately for deconvolution of the voltammetric signals. Chemometrics methods can thus be used for the quantitative analysis of two components in commercial drugs with simple operation and satisfactory results.

## Figures and Tables

**Table 1:** The parameters of linear equation obtained by the proposed method.

Parameters	Pyridoxine	Hydrochlorothiazide
Sample number (*n*)	11	11
Linear range (μg/mL)	0.02–0.40	0.10–2.0
Intercept (nA)	14.38	12.12
Slope(nA∙mL/μg)	0.60	0.20
Correlation coefficient	0.9995	0.9995
Limit of detection (ng/mL)	7.00	37.60

**Table 2:** Prediction results for pyridoxine(PX) and hydrochlorothiazide(HC) validation samples by different chemometrics methods (μg/mL).

Sample	Added		Found (CLS)		Found (PLS) ^1^		Found (PCR)^2^	
	PX	HC	PX	HC	PX	HC	PX	HC
1	0.025	0.100	0.212	0.322	0.025	0.173	0.024	0.143
2	0.025	0.275	0.194	0.418	0.026	0.260	0.027	0.187
3	0.050	0.600	0.150	0.641	0.041	0.572	0.043	0.506
4	0.050	0.900	0.111	0.834	0.049	0.891	0.049	0.840
5	0.110	0.100	0.239	0.195	0.133	0.068	0.134	0.057
6	0.110	0.275	0.210	0.372	0.105	0.272	0.106	0.251
7	0.110	0.600	0.173	0.558	0.113	0.563	0.114	0.542
8	0.110	0.900	0.141	0.759	0.101	0.888	0.101	0.843
9	0.170	0.100	0.255	0.162	0.188	0.811	0.189	0.096
10	0.170	0.275	0.228	0.303	0.173	0.264	0.174	0.278
11	0.170	0.600	0.194	0.508	0.164	0.568	0.165	0.552
12	0.170	0.900	0.156	0.680	0.162	0.873	0.162	0.858
RPE_S_ (%)^3^			51.8	23.2	8.18	5.47	8.41	9.33
Recovery (%)^4^			277.4	134.4	100.1	99.19	100.5	91.58
RPE_T_ (%)^3^			46.8		7.04		8.86	

^1^ The factors of PLS is 4. ^2^ The factors of PCR is 4. ^3^ RPE_S_ (%) and RPE_T_ (%) are relative prediction errors for single and total components, respectively. ^4^ Recovery (%) = 100 × ∑ (c_ij,pred_ − c_ij,added_)/*n*, where n is the number of samples, c_ij_ is the concentration of the *j-*th component in the *i-*th sample.

**Table 3:** Pyridoxine(PX) and hydrochlorothiazide(HC) in commercial tablets as determined by the proposed method (mg/tablet).

Sample	Target values		Found by this method		Recovery(%)	
	PX	HC	PX	HC	PX	HC
Tablet 1^1^	0.5	1.6	0.47 ± 0.03	1.48 ± 0.02	94.0	92.5
Tablet 2^2^	0.5	1.6	0.44 ± 0.04	1.69 ± 0.03	88.0	105.6
Tablet 3^3^	1.0	3.1	1.01 ± 0.02	3.29 ± 0.01	101.0	106.1
Tablet 4^4^	1.0	3.1	1.04 ± 0.02	3.25 ± 0.03	104.0	104.8

^1^ Jinhua Yexing Pharmaceutical Co., Ltd., Shanxi. Lot code. 061108. ^2^ Dongzhitang Pharmaceutical Co., Ltd., Anhui. Lot code. 06101102. ^3^ Yinhe Pharmaceutical Factory, Jilin. Lot code. 20050801. ^4^ Fenhe Pharmaceutical Co., Ltd., Shanxi. Lot code. 0701031.
